# Mycotoxin Occurrence and Microbiological Quality of Maize Silage Supplemented with Insect Meals for Potential Use in Waterfowl Nutrition

**DOI:** 10.3390/ani16152386

**Published:** 2026-08-03

**Authors:** Matúš Džima, Miroslava Kačániová, Daniel Bíro, Milan Šimko, Branislav Gálik, Michal Rolinec, Ondrej Hanušovský, Miroslav Juráček

**Affiliations:** 1 Department of Animal Nutrition, Institute of Nutrition and Genomics, Faculty of Agrobiology and Food Resources, Slovak University of Agriculture in Nitra, Tr. A. Hlinku 2, 94976 Nitra, Slovakia; xdzima@uniag.sk (M.D.); daniel.biro@uniag.sk (D.B.); milan.simko@uniag.sk (M.Š.); branislav.galik@uniag.sk (B.G.); michal.rolinec@uniag.sk (M.R.); 2 Institute of Horticulture, Faculty of Horticulture and Landscape Engineering, Slovak University of Agriculture in Nitra, Tr. A. Hlinku 2, 94976 Nitra, Slovakia; miroslava.kacaniova@uniag.sk; 3 School of Medical & Health Sciences, VIZJA University, Okopowa 59, 01043 Warszawa, Poland

**Keywords:** yellow mealworm, black soldier fly, corn silage, urea, lauric acid, silage additives, feed safety, poultry nutrition, fermented feed

## Abstract

Whole crop maize silage may represent a complementary feed material in waterfowl nutrition, but its hygienic quality and mycotoxin profile remain important safety considerations. This study evaluated the microbiological quality, occurrence, and concentrations of selected mycotoxins in non-inoculated whole crop maize silage under five treatments: untreated control (CON), urea-supplemented silage (UR), silage supplemented with full fat *Hermetia illucens* meal (HI), silage supplemented with full fat *Tenebrio molitor* meal (TM), and lauric acid-supplemented silage (LA). All the variants were prepared from freshly harvested chopped maize forage and ensiled for 8 weeks in three independent replicates per treatment. The silages were analyzed for microbiological indicators and for the occurrence of fumonisin B1 (FB1), fumonisin B2 (FB2), total aflatoxins (ΣAFs), ochratoxin A (OTA), zearalenone (ZEA), T2 toxin (T2), HT2 toxin (HT2) and deoxynivalenol (DON). The initial occurrence of the selected mycotoxins in the raw materials was also assessed. In the silages, FB1 and FB2 were detected only in CON and UR, whereas both the fumonisins remained below the limit of detection in HI, TM and LA. DON was detected in all the treatments; HI, TM and CON did not differ significantly and showed the highest concentrations, whereas LA and UR were significantly lower and also differed from each other, with the lowest concentration observed in UR. HT2 was detected in CON and in one TM sample, while T2, ΣAFs, OTA and ZEA remained below the limit of detection in all the silage treatments. No significant differences were observed among the treatments in total viable count, lactic acid bacteria, coliform bacteria or microscopic filamentous fungi, and all the monitored microbiological indicators remained at low levels. Overall, the tested additives did not produce a uniform reduction across the monitored mycotoxin profile. A lower fumonisin occurrence was observed in HI, TM and LA, which should be interpreted with caution given the limited replication, whereas DON remained present in all the treatments.

## Simple Summary

Whole crop maize silage may be used as a complementary feed material for geese and ducks, but its safety depends on its microbiological quality and mycotoxin contamination. In this study, maize silage was prepared without a biological inoculant and supplemented with *Hermetia illucens* meal, *Tenebrio molitor* meal, urea and lauric acid. The aim was to evaluate whether these additives influenced the microbiological quality and mycotoxin profile of the silage. Fumonisins were detected only in the untreated and urea-treated silages, whereas they remained below the limit of detection in the silages supplemented with insect meals or lauric acid. Deoxynivalenol was present in all the treatments and was not uniformly reduced by the additives. The monitored microbiological indicators remained at low levels and did not differ among the treatments. These findings suggest that insect meals and lauric acid may selectively affect fumonisin occurrence in maize silage, but they do not provide a general reduction in all the monitored mycotoxins.

## 1. Introduction

Whole crop maize silage has been evaluated as a feed component in waterfowl nutrition, particularly in geese and broiler ducks. Geese can digest fibrous feed materials at a higher rate than many other poultry species, which is associated with their well-developed caecum and large intestine involved in microbial degradation, as well as a strongly muscular gizzard capable of generating higher mechanical pressure [1]. In White Kołuda W31 geese, partial replacement of a commercial diet with whole crop maize silage reduced the body weight at the end of the rearing period, but the final body weight after oat fattening did not differ significantly, and the feeding system was considered economically favorable because of the lower rearing costs [2]. In Turkish indigenous geese, dietary maize silage inclusion up to 40% did not affect the growth performance, the dressing percentage, the liver and gizzard weights, the digestive system length or the organoleptic meat quality [1]. In Holdobaki geese, a 30% whole-plant maize silage treatment showed the highest economic benefit, whereas a 50% replacement level significantly reduced the final body weight and the average daily gain and increased the feed conversion ratio [3]. In broiler ducks, restricted commercial feeding combined with ad libitum whole-plant maize silage improved the feed conversion ratio and the estimated economic profit without adverse effects on the carcass characteristics [4]. These findings indicate that maize silage may be considered a complementary feed material in waterfowl nutrition, provided that its inclusion level is adjusted to the feeding strategy and the overall diet formulation.

However, maize silage is characterized by a relatively low crude protein content. Increasing its crude protein concentration through suitable nitrogen-rich additives may therefore improve its compositional value and make it more suitable as a complementary feed material in waterfowl diets where part of the concentrate feed is replaced by silage. Insects are increasingly discussed as alternative protein sources in poultry and pig nutrition because of their favorable nutrient composition and potential functional properties [5,6]. In poultry, *Tenebrio molitor* (yellow mealworm) meal has already been evaluated in broiler and layer diets, where low dietary inclusion levels did not deteriorate the meat quality, the feed microbial stability or the selected intestinal morphology parameters [7,8]. In the context of maize silage, insect meals may increase the crude protein and fat content and, through bioactive components, may also affect the microbial development and the mycotoxin occurrence during ensiling [9,10]. This is particularly relevant for *Hermetia illucens* (black soldier fly), which is characterized by a high proportion of medium-chain fatty acids, especially lauric acid [9,11]. Insect components such as chitin, chitosan and antimicrobial peptides have been associated with antimicrobial- or microbiota-modulating activity [9,12,13]. In a recent maize silage study with a lactic acid bacteria (LAB) inoculant, *H. illucens* and *T. molitor* meals improved the selected compositional parameters, while the monitored microbial indicators were not significantly affected and only selective changes were observed in the deoxynivalenol (DON) and fumonisin B1 (FB1) concentrations [10].

The monitored mycotoxins are produced by fungi commonly associated with cereal crops and stored feed materials. DON, zearalenone (ZEA), fumonisins (FUMs), T2 toxin (T2) and HT2 (HT2) toxin are mainly associated with *Fusarium* spp. [14], aflatoxins (AFs) with *Aspergillus* spp. [15], and ochratoxin A (OTA) with both *Aspergillus* and *Penicillium* spp. [16]. Maize silage is frequently contaminated with *Fusarium* mycotoxins, particularly deoxynivalenol and fumonisins. Kaur et al. [17] detected DON in all their analyzed silage maize samples, while the survey data confirmed DON among the most frequent mycotoxins in maize silage and fumonisins as prevalent contaminants that often occur together with other mycotoxins [18,19]. The selected mycotoxins are relevant to poultry and waterfowl safety because fumonisins, DON and ZEA reduced the body weight gain and increased the feed conversion ratio in ducks when administered together, although the individual toxins did not impair performance under the same conditions [20]. DON is mainly relevant through intestinal effects, including altered intestinal morphology, reduced expression of genes involved in tight junction integrity and changes in nutrient transporters in broilers [21]. AFs, particularly aflatoxin B1 (AFB1), are of special concern in ducks because of their high hepatic conversion of AFB1 into AFB1 dihydrodiol, which may explain the animals’ high susceptibility to AFB1 toxicity [22]. In ducks, AFB1 also reduced growth performance, disturbed antioxidant status and induced liver cholestasis through changes in genes involved in bile acid metabolism [23]. OTA-contaminated maize reduced the average daily gain, increased the feed-to-gain ratio and impaired the intestinal barrier and the mitochondrial function in young ducks [24]. T2 inhibited growth and feed intake in ducks and induced hepatic steatosis, lipid metabolism disruption and oxidative stress in the liver [25].

Therefore, the aim of this study was to evaluate the microbiological quality and the occurrence and concentrations of the selected mycotoxins in whole crop maize silage prepared without inoculation and supplemented with *H. illucens* meal or *T. molitor* meal, urea, and lauric acid as comparative treatments in the context of their potential use in waterfowl nutrition.

## 2. Materials and Methods

### 2.1. Sample Preparation

Whole crop maize, hybrid Kathedralis (*Zea mays* L., FAO 480, dent type) was used as the ensiling material. Kathedralis is a late two-line stay-green silage maize hybrid suitable for maize and sugar beet production areas. The maize plants were obtained in cooperation with PD Kozárovce, located in the Levice District, western Slovakia (48°18′47.7″ N, 18°30′31.6″ E). This hybrid was selected because it was grown under the conditions of the cooperating farm and was considered suitable for the local growing environment.

The maize biomass was harvested manually at the milk-to-dough stage by cutting representative plants in the field. The harvested plants were transported to the laboratory and mechanically chopped using a shredder (Hecht 6284 XL, WERCO spol. s r.o., Prague, Czech Republic) to a theoretical length of cut of 20 mm. The whole chopped batch was thoroughly mixed on a clean surface and then divided into equal portions, from which the individual treatments and the samples of the fresh forage were taken. The chopped material was used immediately for laboratory scale ensiling. The dry matter content of the fresh maize biomass at harvest was 32.52%. A representative sample of the chopped maize material was collected before ensiling for chemical characterization. No biological silage inoculant was applied during harvesting, chopping, or preparation of the experimental treatments.

The laboratory ensiling experiment was carried out in the Laboratory of Feed Conservation, Department of Animal Nutrition, Institute of Nutrition and Genomics, Faculty of Agrobiology and Food Resources, Slovak University of Agriculture in Nitra. All experimental variants were prepared from the same batch of manually harvested and chopped maize biomass.

Five treatments were prepared, each in three independent replicates, giving a total of 15 independently ensiled experimental units (5 treatments × 3 replicates). Each replicate consisted of a separate vacuum bag that was filled, sealed, stored and opened individually, and each bag represented one experimental unit for all subsequent analyses. The treatments consisted of untreated maize forage as the control (CON), maize forage supplemented with urea (UR), maize forage supplemented with full fat *Hermetia illucens* meal (HI), maize forage supplemented with full fat *Tenebrio molitor* meal (TM), and maize forage supplemented with lauric acid (LA). Urea was included as a conventional silage additive commonly used to increase the crude protein content of maize silage. The amount of nitrogen supplied by urea was used as the reference basis for calculating the inclusion levels of *H. illucens* and *T. molitor* meals. Lauric acid was included as a comparative additive because it is a characteristic fatty acid of *H. illucens*. Whole dried *H. illucens* larvae and whole dried *T. molitor* larvae were purchased as commercially available products (Slovakia). Lauric acid (CAS 143-07-7) was also purchased as a commercially available product (Czech Republic). According to EU legislation, urea is authorized only for ruminants with a functional rumen and is therefore not authorized for poultry, whereas lauric acid is authorized as a sensory additive within the functional group of flavoring compounds for all animal species [26,27,28].

Urea was added at 0.3% of fresh matter, corresponding to 3 g/kg fresh matter and 1.35 g N/kg fresh matter, assuming that urea contained 45% N. The inclusion rates of the insect meals were calculated from their analyzed crude protein concentrations using a nitrogen-to-crude-protein conversion factor of 6.25. The resulting nitrogen concentrations were 53.63 g/kg for *H. illucens* meal and 69.79 g/kg for *T. molitor* meal. Based on the nitrogen supplied by the urea treatment, the inclusion rates were set at 25.17 g/kg fresh matter for *H. illucens* meal and 19.34 g/kg fresh matter for *T. molitor* meal. The lauric acid treatment was formulated to provide an equivalent amount of lauric acid as supplied by the *H. illucens* meal. This calculation was based on the analyzed ether extract and lauric acid content of *H. illucens* meal (Table 1). Total fatty acids were estimated from the ether extract content using a fat-to-fatty-acid conversion factor of 0.95, in line with the lipid conversion factors derived for foods of animal origin [29,30]. The resulting lauric acid equivalent was 1.78 g/kg fresh matter. Before incorporation into the maize biomass, the whole dried larvae were ground through a 1 mm sieve. The maize biomass and insect meals were analyzed before ensiling to determine dry matter, crude protein, ether extract, and lauric acid content.

For each replicate, the respective additive was weighed separately and manually mixed with the chopped maize biomass to obtain a homogeneous mixture. The prepared material was ensiled in 3.5 L plastic vacuum bags. Each bag was filled with 0.85 ± 0.05 kg of fresh chopped material (range 0.76–0.97 kg), corresponding to a packing density of 244 ± 14 kg of fresh matter per m^3^. The bags were sealed using a vacuum sealer (MSW Motor Technics, Berlin, Germany) and stored at 21 °C for 8 weeks. After the storage period, the silage units were opened, and representative samples were collected from each replicate. One representative sample was taken from each of the three bags per treatment. Samples intended for chemical analysis were pre-dried at 55 ± 5 °C and ground to pass through a 1 mm sieve using a laboratory mill (Fritsch, Idar-Oberstein, Germany).

The chemical composition of used materials was determined in the Laboratory of Feed Quality and Nutritional Value, Institute of Nutrition and Genomics, Faculty of Agrobiology and Food Resources, Slovak University of Agriculture in Nitra, using standard procedures [31].

### 2.2. Mycotoxin Analysis

For mycotoxin analysis, three independent subsamples of each insect meal and of the fresh chopped maize forage were taken from the respective homogenized batches and analyzed separately (Table 2). Silage mycotoxin concentrations were determined in three independent samples per treatment, each originating from a separate ensiled bag. The analysis of selected mycotoxins was performed in cooperation with the accredited laboratory Romer Labs Diagnostic GmbH (Tulln, Austria). The analyzed mycotoxins included FB1, FB2, ΣAFs, OTA, ZEA, T2, HT2, and DON. ΣAFs were calculated according to the following equation:

ΣAFs = AFB1 + AFB2 + AFG1 + AFG2(1)

Mycotoxin determination was carried out by HPLC-MS/MS using the Romer Labs procedure AT SOP 31 (Romer Labs Diagnostic GmbH, Tulln, Austria), which is based on EN 17280:2019 [32]. A total of 10 g of each sample was extracted with 30 mL of acetonitrile/water (7:3, *v*/*v*). After centrifugation, the extract was diluted tenfold with HPLC eluent A. Internal standards were added before dilution. Chromatographic separation was performed on a Phenomenex Gemini C18 column (4.6 × 150 mm, 5 µm; Phenomenex Inc., Torrance, CA, USA) using an Agilent 1260 Infinity II HPLC system (Agilent Technologies, Santa Clara, CA, USA) coupled to MS/MS detection. Quantification was performed by internal standard calibration using calibration curves based on analyte-to-internal standard signal ratios. Identification was conducted according to SANTE 12089/2016 [33], using retention time and two product ions as identification criteria. Two internal control samples were included in each analytical batch. The limits of detection (LODs) were 0.3 µg/kg for AFB1, 0.5 µg/kg for AFB2, AFG1, and AFG2, 15 µg/kg for DON and HT2, 10 µg/kg for FB1, FB2, and T2, and 3 µg/kg for ZEA. Mycotoxin concentrations were initially determined on a laboratory dry matter basis and subsequently recalculated to 88% dry matter, with results expressed as µg/kg.

### 2.3. Microbiological Analyses

One silage sample from each of the three bags per treatment (n = 3) was subjected to microbiological analysis. The assessment included the enumeration of total viable microorganisms (TVCs), lactic acid bacteria (LAB), coliform bacteria (CB), and microscopic filamentous fungi (MFF; Table 3). For the initial suspension, 5 g of each silage sample was aseptically transferred into 45 mL of sterile 0.87% saline solution and homogenized. Decimal serial dilutions ranging from 10^−2^ to 10^−4^ were then prepared.

TVCs were enumerated by spreading 100 µL of the appropriate dilution on Tryptic Soy Agar (TSA; Sigma-Aldrich^®^, St. Louis, MO, USA), followed by incubation at 30 °C for 48–72 h. CB were determined on MacConkey agar (MC; Sigma-Aldrich^®^, St. Louis, MO, USA) after incubation at 37 °C for 24–48 h. LAB were cultivated on MRS agar (Sigma-Aldrich^®^, St. Louis, MO, USA) under microaerophilic conditions with 5% CO_2_ at 37 °C for 72 h. MFF were enumerated on malt extract agar (MEA; Sigma-Aldrich^®^, St. Louis, MO, USA) supplemented with bromocresol green at 0.020 g/L and incubated aerobically at 25 °C for 5 days. The results were expressed as colony forming units (CFUs) and converted to log CFU/g.

### 2.4. MALDI-TOF MS Biotyper Identification of Microbial Isolates

Microbial isolates obtained from microbiological analyses were identified by matrix-assisted laser desorption/ionization time-of-flight mass spectrometry (MALDI-TOF MS Biotyper; Bruker Daltonics, Bremen, Germany) using a Microflex LT/SH Biotyper (Bruker Daltonics, Bremen, Germany). Fresh colonies were subjected to the standard ethanol/formic acid extraction procedure according to the manufacturer’s instructions. The resulting protein extracts were spotted onto a polished steel target plate, overlaid with 1 µL of α-cyano-4-hydroxycinnamic acid (HCCA) matrix solution, and allowed to air-dry at room temperature. Mass spectra were acquired in linear positive-ion mode (*m*/*z* 2000–20,000) and analyzed using MBT Compass software (Bruker Daltonics, Bremen, Germany) against the manufacturer’s reference database. Identification scores below 1.700 were considered unreliable, scores between 1.700 and 1.999 indicated probable genus identification, scores between 2.000 and 2.299 indicated secure genus identification with probable species identification, and scores between 2.300 and 3.000 indicated highly probable species identification. A score-oriented dendrogram was generated to visualize the relatedness of the mass spectral profiles among isolates recovered from all treatment groups.

### 2.5. Statistical Analysis

Statistical evaluation was performed in IBM SPSS Statistics 26.0 (IBM Corp., Armonk, NY, USA). Concentrations below the limit of detection (<LOD) were not replaced by substituted values such as zero, LOD/2 or LOD/√2. Means and standard deviations are therefore based on the samples in which the respective mycotoxin was detected, and the proportion of positive samples is reported separately as the incidence. Where a mycotoxin was detected in a single sample of a treatment, the individual value is reported without a standard deviation. Differences among treatments were tested using one-way analysis of variance, with Tukey’s HSD test applied for post hoc multiple comparisons. The assumptions of the parametric model were assessed on the model residuals. Normality was evaluated by the Shapiro–Wilk test (*p* ≥ 0.16 for all analyzed variables) and homogeneity of variances by Levene’s test based on the median (*p* ≥ 0.65 for all analyzed variables). With three replicates per treatment, formal testing of these assumptions has limited power. The treatment effects were therefore additionally verified by the non-parametric Kruskal–Wallis test, which confirmed the outcome of the one-way ANOVA for all analyzed variables. Welch’s ANOVA gave the same result for DON (F = 361.7; *p* < 0.001). Results are expressed as mean ± standard deviation (SD), and differences were considered statistically significant at *p* < 0.05.

## 3. Results and Discussion

### 3.1. Mycotoxins

The mycotoxin occurrence in the materials used differed among the tested substrates (Table 2). Fresh maize forage contained detectable levels of FB1, T2, HT2, and DON, with DON showing the highest concentration among the detected mycotoxins. In both the insect meals, most of the monitored mycotoxins were below the limit of detection. The only exception was total aflatoxins, which were detected in two of the three *T. molitor* meal samples.

Among the silage treatments (Table 4), FB1 and FB2 were detected only in CON and UR, without significant differences (*p* > 0.05) between these treatments, whereas FUM remained below the limit of detection in HI, TM and LA. FB1 concentrations in CON and UR were higher than the concentration measured in fresh maize forage. This increase is most likely related to the heterogeneous, uneven distribution of *Fusarium* mycotoxins in the field material and to limited fungal activity during the initial phase of ensiling, rather than to a synthesis under stable anaerobic conditions [34]. HT2 was detected in CON and in one of the three TM samples, whereas T2, total aflatoxins, OTA and ZEA remained below the limit of detection in all the silage treatments. T2 was detected in fresh maize forage but not in the silages. Since HT2 is a deacetylated metabolite of T2, this pattern may be compatible with the partial transformation of T2 during ensiling [35,36]. However, this conversion was not directly assessed in the present study. DON was detected in all the silage treatments. Its highest concentrations were observed in HI, TM and CON, which did not differ significantly (*p* > 0.05) from each other, followed by LA, whereas the lowest concentration occurred in UR and differed significantly (*p* < 0.05) from all the other treatments. DON concentrations were higher in all the silages than in the fresh maize forage.

The higher DON concentrations in the silages may partly reflect the release of free DON from its masked forms during early ensiling. Deoxynivalenol-3-glucoside is one masked form and can be converted to free DON, thereby increasing the concentration of the free DON quantified in the present study [37]. Since only free DON was quantified, DON 3 glucoside in fresh forage could have contributed to the apparent increase in free DON after fermentation. In addition, the first weeks of ensiling, before stable anaerobic conditions are established, may still permit limited activity of field-derived *Fusarium* spp. and further toxin formation [34,37]. The pronounced variability of DON in the fresh forage (461.53 ± 472.09 µg/kg) also indicates the heterogeneous and uneven contamination of the field material, which can contribute to the differences between the analyzed forage and silage subsamples.

Maize silage is frequently contaminated with *Fusarium* mycotoxins, particularly DON and FUM. Kaur et al. [17] reported DON in all their analyzed silage maize samples, and a recent European survey identified DON as the most prevalent mycotoxin in maize silage, with an occurrence of 97%, while maize silage was also characterized by a high degree of mycotoxin co-occurrence [19]. FUMs are also relevant contaminants of maize silage. Weaver et al. [18] reported that FUMs belong among the prevalent *Fusarium* mycotoxins in maize silage and frequently co-occur with other mycotoxins, while Lapris et al. [38] confirmed their presence in maize silage from northern Italy. Type A trichothecenes may also occur in maize silage, although usually at a lower prevalence than DON. Zhang et al. [39] reported T2 and HT2 in 1.5% and 4.0% of maize silage samples from China, respectively, with HT2 occurring more frequently and at a higher concentration than T2. The present results are consistent with this general contamination pattern, as DON was detected in all the silage treatments, FB1 and FB2 were detected in CON and UR, and HT2 occurred only sporadically, whereas T2 remained below the limit of detection in all the silages. The occurrence of these mycotoxins may reflect contamination established before harvest, while storage conditions and ensiling dynamics can further influence their levels [34].

Kalúzová et al. [40] discussed the potential of urea to influence mycotoxin occurrence during ensiling through partial degradation to ammonia, which may create a more alkaline environment with antifungal activity. However, this expected protective effect was not confirmed by their results, as the changes in mycotoxin concentrations after the additive application were not statistically significant, and the highest concentrations of DON and beauvericin were observed in the urea-treated silage. UR showed the lowest DON concentration, which differed significantly (*p* < 0.05) from all the other treatments. This response may be related to the partial hydrolysis of urea to ammonia, which can create conditions less favorable to residual fungal activity. As pH, ammonia-N and fermentation acids were not evaluated in the present manuscript, this explanation remains a hypothesis that cannot be verified using the data reported here. The lower DON in LA may relate to free lauric acid, which is antifungally active [41], whereas in HI, lauric acid is mainly present in the lipid fraction as an esterified fatty acid in triglycerides, and its availability would depend on lipolytic release [11,42]. TM, low in lauric acid, showed no comparable reduction. As FUMs were not similarly reduced, the effect was not uniform across the mycotoxins.

The absence of detectable FB1 and FB2 in HI, TM and LA may indicate a selective fumonisin response to these additives under the present experimental conditions. However, given the heterogeneous contamination of the field material and only three subsamples per treatment, this fumonisin pattern should be interpreted with caution. Differences in lipid composition and other bioactive components of the additives may have contributed to this response [43,44,45,46]. Nevertheless, the present data does not allow the identification of the exact mechanism. The absence of detectable FB1 and FB2 in HI, TM and LA was not accompanied by significant differences (*p* > 0.05) in the total counts of microscopic filamentous fungi (MFF). This indicates that the fumonisin response was not associated with a measurable reduction in total MFF counts [35,47].

The present findings can be directly compared with the recent maize silage study of Džima et al. [10], which used the same experimental design. The main difference between the two ensiling models was the presence of the LAB-based biological additive. In the LAB-treated model, lower FB1 concentrations were observed in the LAB + *T. molitor* and LAB + lauric acid treatments, whereas LAB + urea and LAB + *H. illucens* did not differ from the inoculated control. In the present silages without the biological additive, both FB1 and FB2 remained below the limits of detection in HI, TM and LA, while they were detected in CON and UR. DON showed a different pattern. In the LAB-treated model, lower DON concentrations were observed in the LAB + urea, LAB + *H. illucens* and LAB + *T. molitor* treatments compared with the inoculated control, whereas in the present experiment, DON was detected in all the treatments, with the highest concentrations in HI, CON and TM, which did not differ significantly (*p* > 0.05), whereas LA and especially UR showed significantly (*p* < 0.05) lower concentrations. These contrasting responses indicate that the effect of the tested additives on mycotoxin occurrence depended strongly on the ensiling model and differed among the individual mycotoxins. Since both the studies used the same maize material and additive concept, the presence of the LAB-based biological additive was probably an important factor contributing to the different DON and fumonisin responses.

From the perspective of feed safety, the detected mycotoxin concentrations were compared with the reference values used in the European Union, the United States and China.

DON was the predominant mycotoxin in the tested silages, with the highest concentration observed in HI at 2.49 mg/kg. This value was below the European guidance value of 5 mg/kg for compound feed [48], below the FDA advisory level of 5 mg/kg for grains and grain byproducts intended for animal categories not specifically listed [49] and below the Chinese limit of 5 mg/kg for feed ingredients of plant origin [50]. It was also below the Chinese limit of 3 mg/kg for other compound feeds [50]. However, the FDA advisory level applies to grains and grain byproducts, and the European and Chinese compound feed values apply to final feed rather than to maize silage as a single dietary component.

The highest measured concentration of FB1 plus FB2 was 0.27 mg/kg. This value was far below the European guidance value of 20 mg/kg for poultry compound feed [48] and below the Chinese limits of 60 mg/kg for maize silage and 20 mg/kg for poultry compound feed [50]. It was also far below the FDA recommended maximum levels for total FUMs in corn and corn byproducts intended for poultry [51].

T2 remained below the limit of detection in all the silage treatments, whereas HT2 reached 261.85 µg/kg in CON. The European Union provides indicative levels of 250 µg/kg for the sum of T2 and HT2 in compound feed other than feed for cats and 500 µg/kg for other cereal products intended for feed [52]. The CON value was therefore slightly above the indicative level for compound feed but below the indicative level for other cereal products intended for feed. The Chinese standard provides a limit of 0.5 mg/kg for T2 in feed ingredients of plant origin and in pig and poultry compound feeds, but not for HT2 or for the sum of T2 and HT2 [50]. The FDA has not established an advisory or recommended maximum level for T2 or HT2 in animal feed.

The study was conducted at laboratory scale with three independently ensiled units per treatment, which limits statistical power, particularly given the heterogeneous mycotoxin contamination of the original maize material, as indicated by the high variability of DON in fresh forage (461.43 ± 472.09 µg/kg; Table 2). Therefore, the observed treatment differences, especially the absence of detectable fumonisins in HI, TM and LA, should be interpreted as indicative rather than conclusive. Confirmation will require a larger number of independent silage units and more uniformly contaminated material. Basic fermentation characteristics, including pH, ammonia-N and organic acids, were not included in the present manuscript, which limits the interpretation of the proposed mechanisms and microbiological results.

### 3.2. Microbiological Indicators

No significant differences (*p* > 0.05) in TVC, LAB, CB, or MFF were observed among the treatments (Table 5). These findings indicate that the tested additives did not significantly affect the selected microbiological parameters of the maize silages. The recorded values were well below the indicative reference limits reported for the microbiological quality assessment of feed, namely 6.48 log CFU/g for TVC, 4.00 log CFU/g for CB, and 5.30 log CFU/g for MFF [53,54]. Therefore, none of the monitored microbial groups indicated an impaired microbiological quality of the tested maize silages.

Similarly low microbial counts were reported by Džima et al. [10] in maize silage treated with LAB inoculants. In that study, the monitored microbiological parameters also remained low and did not differ significantly among the treatments. In contrast, Kalúzová et al. [40] reported higher TVC and MFF counts in untreated, LAB-treated, and urea-treated maize silages than those observed in the present study, whereas CB remained undetectable in all the treatments. Other studies have shown that LAB inoculation may increase LAB populations or suppress yeasts and filamentous fungi under specific conditions; however, these effects appear to depend on the silage characteristics and the experimental conditions and are therefore not consistently observed across studies [55,56].

**Figure 1:**
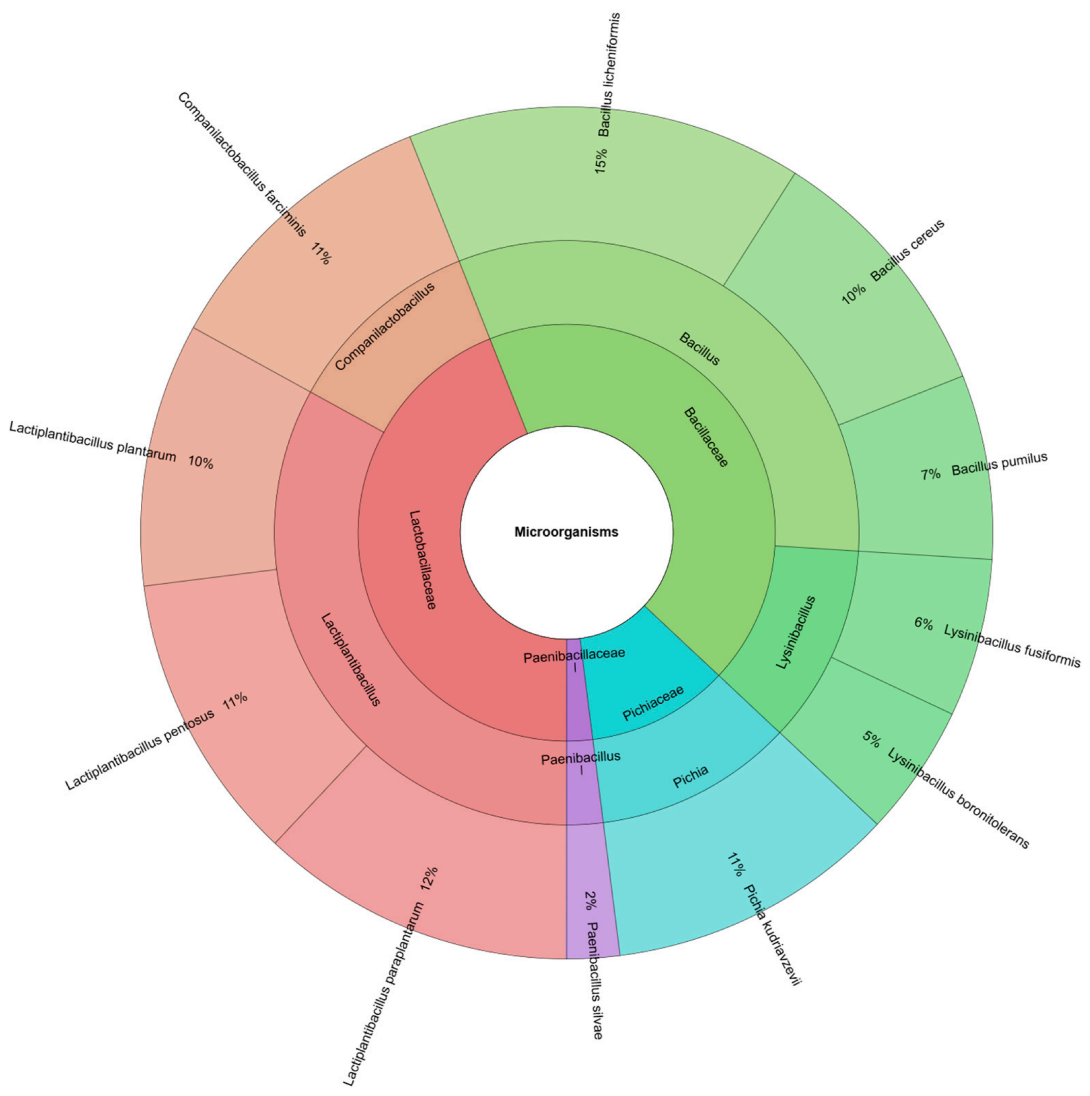
Taxonomic composition of microbial isolates from all treatment groups.

A total of 215 microbial isolates were identified by MALDI-TOF MS, representing 11 species belonging to four families (Figure 1). The predominance of members of the families *Lactobacillaceae* and *Bacillaceae* observed in the present study is consistent with the microbial communities commonly reported in maize silages. Members of *Lactobacillaceae*, including *Lactiplantibacillus* spp. and *Companilactobacillus farciminis*, are typical lactic acid bacteria involved in silage fermentation and contribute to acidification and preservation through lactic acid production [57]. In contrast, species of *Bacillus* and *Lysinibacillus* are ubiquitous spore-forming bacteria commonly associated with plant material and silage that are able to survive the ensiling process in a dormant state. *Pichia kudriavzevii*, representing 11% of the identified isolates, has been described as one of the principal yeasts involved in aerobic deterioration of silage because it can assimilate lactic acid after air exposure, thereby increasing the silage pH [58]. However, the low counts of spoilage microorganisms observed in the present study suggest that the overall microbiological quality of the analyzed silages remained favorable.

Table 6 shows the presence (+) or absence (−) of the individual identified microbial species across the experimental groups (CON, UR, HI, TM, and LA). Most of the identified species, namely *B. cereus*, *B. licheniformis*, *C. farciminis*, *L. paraplantarum*, *L. pentosus*, *L. plantarum*, and *P. kudriavzevii*, were detected in all five groups regardless of the additive used. In contrast, the occurrence of *B. pumilus*, *L. boronitolerans*, *L. fusiformis,* and *P. silvae* varied among the groups, suggesting that the individual additives may have influenced the qualitative composition of the cultivable microbial community.

## 4. Conclusions

Regardless of the treatment applied, DON was the predominant mycotoxin in the maize silages and was detected with 100% incidence. FUM remained below the limit of detection in HI, TM, and LA, whereas both the toxins were detected in CON and UR. This suggests a selective fumonisin response to the insect meals and lauric acid under the present experimental conditions. In contrast, DON was detected in all the silage treatments. The highest DON concentrations were observed in HI, TM and CON, which did not differ significantly (*p* > 0.05), whereas the lowest concentration was found in UR. The insect meal treatments therefore did not reduce DON relative to the untreated control, whereas lauric acid and urea were associated with significantly (*p* < 0.05) lower DON concentrations, with urea showing the lowest. HT2 was detected in CON and in one TM sample, while T2, total aflatoxins, OTA and ZEA remained below the limit of detection in all the silage treatments.

None of the tested additives significantly (*p* > 0.05) affected the selected microbiological indicators of the silages. TVC, LAB, CB and MFF remained at low levels in all the treatments and did not indicate impaired microbiological quality. The cultivable microbial isolates identified by MALDI-TOF MS were dominated by members of the families *Lactobacillaceae* and *Bacillaceae*, with *Lactiplantibacillus*, *Companilactobacillus*, and *Bacillus* representing the predominant bacterial genera identified in the maize silages. Therefore, the differences observed in the mycotoxin profile, particularly for FUM and DON, were not directly reflected by changes in the total counts of microscopic filamentous fungi.

These findings indicate that the effects of insect meals, urea and lauric acid on the hygienic quality of maize silage were specific to individual mycotoxins rather than uniform across the whole monitored profile. Insect meals and lauric acid may be associated with a favorable fumonisin response in non-inoculated maize silage, but they did not provide a general reduction in all the monitored mycotoxins. The results should be interpreted within the limits of a laboratory-scale ensiling study in which no feeding trial with waterfowl was conducted, and their practical relevance for waterfowl nutrition requires confirmation in future feeding studies.

## Figures and Tables

**Table 1:** Basic nutritional parameters and lauric acid content of the evaluated feed materials.

	DM	CP	EE	C12:0
	g/kg	g/kg DM	g/kg DM	% of Total FA
Maize forage	325.23 ± 0.19	53.76 ± 1.16	18.79 ± 0.29	N.D.
Black soldier fly * (*H. illucens*)	960.25 ± 0.96	349.05 ± 0.95	340.12 ± 2.32	22.76 ± 0.35
Yellow mealworm * (*T. molitor*)	952.55 ± 1.12	457.92 ± 1.79	311.01 ± 7.92	0.34 ± 0.01

Abbreviations: DM—dry matter, CP—crude protein, EE—ether extract, C12:0—lauric acid, N.D.—not detected; * DM, CP, EE and C12:0 values for *H. illucens* and *T. molitor* were previously published in Džima et al. [10,13]; values are presented as mean ± SD; n = 3 per material.

**Table 2:** Concentrations and incidence of selected mycotoxins in fresh maize forage and insect materials.

	Maize Forage	Black Soldier Fly (*H. illucens*)	Yellow Mealworm (*T. molitor*)
	µg/kg 88% DM		
FB1	93.62 ± 7.65 (66.66) *	<LOD	<LOD
FB2	<LOD	<LOD	<LOD
ΣAFs	<LOD	<LOD	6.00 ± 6.84 (66.66) *
OTA	<LOD	<LOD	<LOD
ZEA	<LOD	<LOD	<LOD
T2	274.18 ± 104.76 (100) *	<LOD	<LOD
HT2	249.66 ± 116.92 (100) *	<LOD	<LOD
DON	461.43 ± 472.09 (100) *	<LOD	<LOD

Abbreviations: DM—dry matter, FB1—fumonisin B1, FB2—fumonisin B2, ΣAFs—total aflatoxins, OTA—ochratoxin A, ZEA—zearalenone, T2—T2 toxin, HT2—HT2 toxin, DON—deoxynivalenol, <LOD—below the limit of detection; values below the LOD were not included in the calculation of means and SD; means and SD are therefore based on the positive samples only; * values in parentheses show mycotoxin incidence as the percentage of positive samples, 100 indicates detection in all analyzed samples of the respective material (3/3); three independent samples were analyzed for each material.

**Table 3:** Counts of selected microbial groups in fresh maize forage before ensiling.

	TVC	CB	LAB	MFF
	log CFU/g			
Maize Forage	2.31 ± 0.14	2.50 ± 0.17	1.67 ± 0.11	N.D.

Abbreviations: CFU—colony forming unit, TVC—total viable count, CB—coliform bacteria, LAB—lactic acid bacteria, MFF—microscopic filamentous fungi, N.D.—not detected; values are presented as mean ± SD; n = 3.

**Table 4:** Mycotoxin content and incidence of selected mycotoxins in the tested maize silages.

	CON	UR	HI	TM	LA
	µg/kg 88% DM				
FB1	150.79 ± 18.05 (100) *	142.95 ± 12.94 (100) *	<LOD	<LOD	<LOD
FB2	119.31 ± 8.22 (66.66) *	130.70 ± 3.66 (100) *	<LOD	<LOD	<LOD
ΣAFs	<LOD	<LOD	<LOD	<LOD	<LOD
OTA	<LOD	<LOD	<LOD	<LOD	<LOD
ZEA	<LOD	<LOD	<LOD	<LOD	<LOD
T2	<LOD	<LOD	<LOD	<LOD	<LOD
HT2	261.85 ± 103.57 (66.66) *	<LOD	<LOD	133.27 (33.33) *	<LOD
DON	2131.21 ^a^ ±206.94 (100) *	612.37 ^c^ ±45.06 (100) *	2492.86 ^a^ ±61.98 (100) *	2269.14 ^a^ ±112.14 (100) *	1449.89 ^b^ ±290.13 (100) *

Abbreviations: CON—control, UR—maize silage + urea, HI—maize silage + *H. illucens*, TM—maize silage + *T. molitor* meal, LA—maize silage + lauric acid, DM—dry matter, FB1—fumonisin B1, FB2—fumonisin B2, ΣAFs—total aflatoxins, OTA—ochratoxin A, ZEA—zearalenone, T2—T2 toxin, HT2—HT2 toxin, DON—deoxynivalenol, <LOD—below the limit of detection; values below the LOD were not included in the calculation of means and SD; means and SD are therefore based on the positive samples only; * values in parentheses show mycotoxin incidence as the percentage of positive samples, 100 indicates detection in all analyzed samples of the respective treatment (3/3). Within rows, different superscript letters (^abc^) denote statistically significant differences among treatments (*p* < 0.05); three independent samples were analyzed per treatment. For HT2 in TM, only one of three samples was positive; therefore, no SD could be calculated.

**Table 5:** Counts of selected microbial groups in the tested maize silages.

	CON	UR	HI	TM	LA
	log CFU/g				
TVC	1.45 ± 0.11	1.38 ± 0.06	1.41 ± 0.13	1.52 ± 0.22	1.39 ± 0.04
LAB	1.55 ± 0.21	1.49 ± 0.14	1.41 ± 0.08	1.46 ± 0.09	1.38 ± 0.10
CB	1.38 ± 0.06	1.27 ± 0.06	1.44 ± 0.11	1.33 ± 0.10	1.34 ± 0.09
MFF	1.46 ± 0.26	1.45 ± 0.11	1.38 ± 0.06	1.35 ± 0.10	1.42 ± 0.07

Abbreviations: CON—control, UR—maize silage + urea, HI—maize silage + *H. illucens*, TM—maize silage + *T. molitor* meal, LA—maize silage + lauric acid, CFU—colony forming unit, TVC—total viable count, CB—coliform bacteria, LAB—lactic acid bacteria, MFF—microscopic filamentous fungi; no statistically significant differences were observed among treatments (*p* > 0.05); values are presented as mean ± SD; n = 3 per treatment.

**Table 6:** Presence (+) or absence (−) of identified microbial species across the experimental groups.

	CON	UR	HI	TM	LA
*Bacillus cereus*	+	+	+	+	+
*Bacillus licheniformis*	+	+	+	+	+
*Bacillus pumilus*	−	−	−	+	+
*Companilactobacillus farciminis*	+	+	+	+	+
*Lactiplantibacillus paraplantarum*	+	+	+	+	+
*Lactiplantibacillus pentosus*	+	+	+	+	+
*Lactiplantibacillus plantarum*	+	+	+	+	+
*Lysinibacillus boronitolerans*	−	−	+	+	+
*Lysinibacillus fusiformis*	−	+	+	+	+
*Paenibacillus silvae*	+	−	−	−	−
*Pichia kudriavzevii*	+	+	+	+	+

Abbreviations: CON—control, UR—maize silage + urea, HI—maize silage + *H. illucens*, TM—maize silage + *T. molitor* meal, LA—maize silage + lauric acid.

## Data Availability

The data presented in this study are available from the corresponding author on request.
